# A nuclear protein, PfMORC confers melatonin dependent synchrony of the human malaria parasite *P. falciparum* in the asexual stage

**DOI:** 10.1038/s41598-021-81235-2

**Published:** 2021-01-21

**Authors:** Maneesh K. Singh, Giulliana Tessarin-Almeida, Barbara K. M. Dias, Pedro Scarpellli Pereira, Fahyme Costa, Jude M. Przyborski, Celia R. S. Garcia

**Affiliations:** 1 Department of Parasitology, Institute of Biomedical Science, University of São Paulo, São Paulo, 05508-000 Brazil; 2 Department of Clinical and Toxicological Analyses, School of Pharmaceutical Sciences, University of São Paulo, São Paulo, 05508-000 Brazil; 3 Department of Physiology, Institute of Bioscience, University of São Paulo, São Paulo, 05508-090 Brazil; 4 Department of Biochemistry and Molecular Biology (BiMoBi), Interdisciplinary Research Center (iFZ), Justus-Liebig-University, Giessen, Germany

**Keywords:** Fluorescence imaging, Molecular biology, Transcription

## Abstract

The host hormone melatonin is known to modulate the asexual cell-cycle of the human malaria parasite *Plasmodium falciparum* and the kinase PfPK7 is fundamental in the downstream signaling pathways. The nuclear protein PfMORC displays a histidine kinase domain and is involved in parasite cell cycle control. By using a real-time assay, we show a 24 h (h) rhythmic expression of PfMORC at the parasite asexual cycle and the expression is dramatically changed when parasites were treated with 100 nM melatonin for 17 h. Moreover, PfMORC expression was severely affected in PfPK7 knockout (PfPK7^−^) parasites following melatonin treatment. Parasites expressing 3D7^*morc-GFP*^ shows nuclear localization of the protein during the asexual stage of parasite development. Although the PfMORC knockdown had no significant impact on the parasite proliferation in vitro it significantly changed the ratio of the different asexual intraerythrocytic stages of the parasites upon the addition of melatonin. Our data reveal that in addition to the upstream melatonin signaling pathways such as IP_3_ generation, calcium, and cAMP rise, a nuclear protein, PfMORC is essential for the hormone response in parasite synchronization.

## Introduction

Malaria is one of the deadliest infectious diseases in many tropical and subtropical countries. The life cycle of *Plasmodium* is divided between the mosquito vector and the vertebrate host, and the clinical symptoms of malaria are attributed to the asexual growth inside the host erythrocytes. In the vertebrate host erythrocytes, parasites go through extensive morphological and developmental changes, the so called ring, trophozoite, and schizont forms; at the end of the cycle, 16–32 new merozoites/RBC are released to start a new infectious cycle^1^. The asexual replication of *Plasmodium* follows a well-defined periodicity, i.e., it is completed in multiples of approximately 24 h, varying according to the host and parasite species^2^. Circadian rhythms have been widely studied in bacteria, fungi, plants, and animals^3–5^ and are of special concern for pathogens infection because they are believed to modulate disease transmission to another host^6^. In mammals, the endogenous hormone melatonin is associated with the circadian clock of the day-night cycle^7–9^. Melatonin is synthesized and secreted by the pineal gland in the night^10^ but it has been identified to perform multifunctional roles in other organisms as well^11–14^.

Much emphasis has been given to the components involved in host cell egress and/or invasion, but extensive efforts to understand the fundamental aspects of asexual growth have been recently expanded. The asexually replicating parasite rapidly switches between stages from ring to schizont and this process is coordinated by a highly regulated gene expression program^15,16^. Recently, it was shown that human *P. falciparum* and murine *P. chabaudi* parasites exhibit intrinsic periodicity for gene expression^17,18^. We have demonstrated that melatonin, by modulating parasite Ca^2+^ and cAMP levels, plays a key role in modulating the cell-cycle of *P. falciparum* and *P. chabaudi* within erythrocytes^19–21^. We have also found the loss of synchronization in Protein Kinase 7 knockout (PfPK7^−^) parasites in culture in presence of melatonin, thus indicating that the orphan kinase PfPK7 takes part in this melatonin-mediated signal transduction pathway^22^.

The signaling cascade that links the Ca^2+^ and cAMP increases in Plasmodia activated by melatonin to the downstream events that modulate the *Plasmodia* cell cycle have been extensively investigated in recent years. For example, we have shown that melatonin treatment results in the activation of the mitochondria fission genes FIS1, FIS2 and DYN1^23^, a process necessary to form the new merozoites in segmented schizont. More recently, we performed RNAseq analysis in parasites treated with melatonin and cAMP analogue adenosine 3′,5′-cyclic monophosphate N6-benzoyl/PKA activator (6-Bz-cAMP). In *P. falciparum* 3D7 strain, 38 genes were differentially expressed upon melatonin treatment. Most important, several of these genes encode nucleic acid binding proteins, consistent with the effects of melatonin on parasite synchronization^24^. Chip-on-chip data analysis with one of these melatonin targets, PfNf-YB transcription factor, reveals that this protein binds to the promoter region of various genes including an *apetala2* transcription factor (AP2-TF)^25^. The AP2-TFs have been identified in other apicomplexan parasites as well and in *Plasmodium,* they control the expression of various genes involved in invasion, gametocytogenesis, or in sexual stage development^26–30^. Transcriptional regulation by AP2-TFs may require additional protein interaction and these interactions are key to facilitate transcription^28^. Hillier et al., in their protein–protein interaction network, reveals multiple AP2-TFs interactions^31^ and among them, they have identified a previously annotated kelch-domain containing protein^32^ that has typical *microrchidia* (MORC) like protein characteristics.

In this study, we demonstrate a time-dependent induction of PfMORC (PlasmoDB accession number—PF3D7_1468100) expression when *P. falciparum* parasites are treated with melatonin. By expressing a GFP- and HA-tagged version of PfMORC we have observed that PfMORC is localized in the nucleus during the asexual cycle. In an attempt to gain a deeper understanding of the PfMORC function, we have also engineered a glmS self-cleaving ribozyme into the 3′-UTR region of the *morc*-locus. Using this system, we have been able to reduce the expression of PfMORC in the RBC cycle upon glucosamine induction. Of interest, knocking down the protein levels did not lead to any effect on parasite overall growth progression and parasite number in culture, but it significantly perturbed the developmental ratio of asexual parasites in the presence of melatonin. Overall, we found a nuclear protein that controls the asexual cycle of the *P. falciparum *in vitro culture that can be a potential target for malaria disease containment.

## Results

### Expression of PfMORC in asexual stage

**Figure 1:**
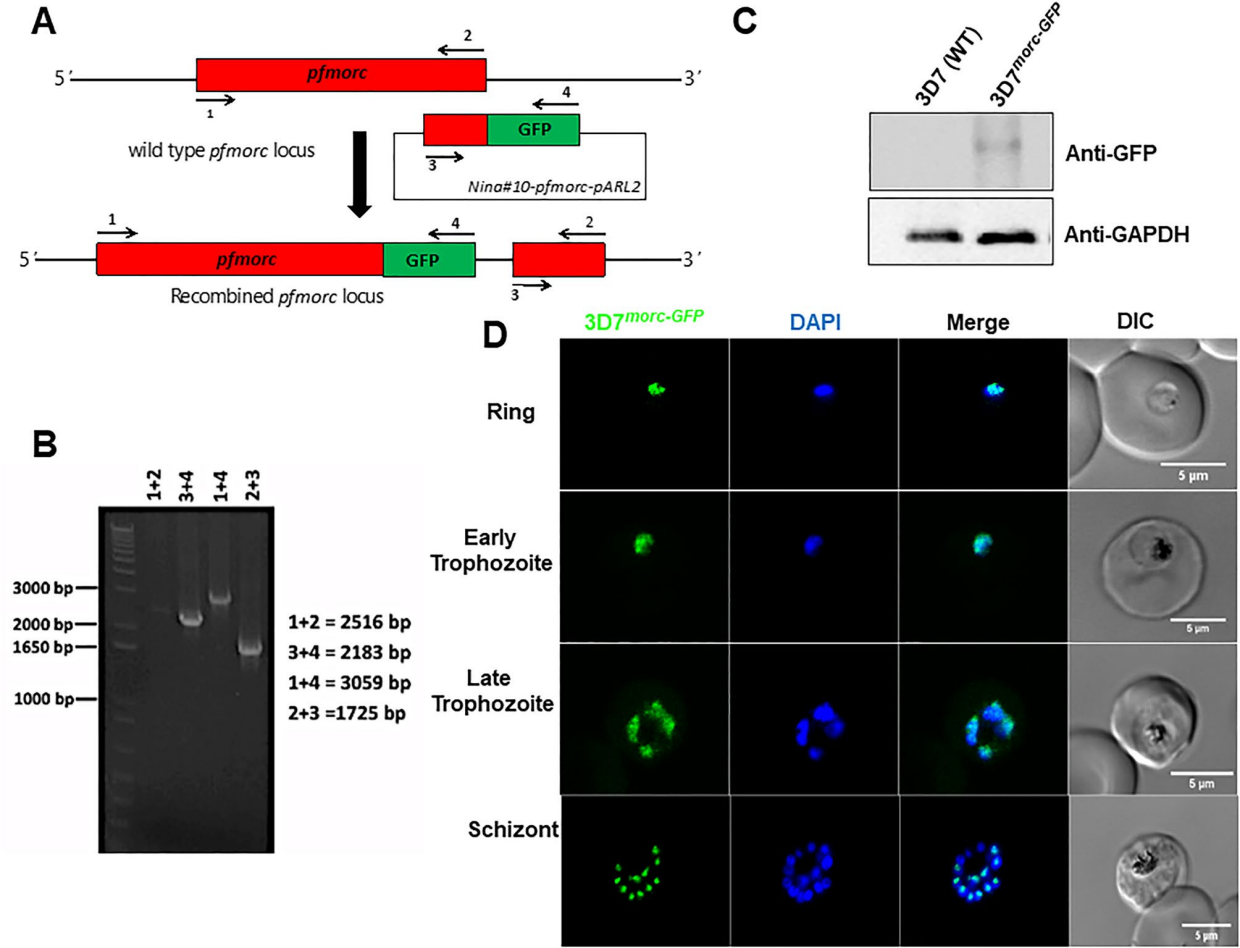
3D7^*morc-GFP*^ construct strategy and localization. (**A**) Schematic representation of the 3D7^*morc-GFP*^ integration strategy and gene after the integration; (**B**) diagnostic PCR; (**C**) Western blot of *P. falciparum* transfected with 3D7^*morc-GFP*^ clones with wild type *P. falciparum* (3D7) showing no band (~ 300 kDa) with anti-GFP antibody, and (**D**) confocal microscopy was performed to localize PfMORC protein in 3D7^*morc-GFP*^-infected erythrocytes. Asexual parasites at ring (6 h.p.i.), early-trophozoite (24 h.p.i.), late-trophozoite (34 h.p.i.) and schizont (44 h.p.i.) stage were used to localize the PfMORC. Parasites were also stained with the nuclear stain DAPI. The colocalization of GFP with the DAPI signal confirms that PfMORC localizes in the nucleus of the asexual developmental stages.

MORC is a highly conserved nuclear protein that exerts a wide range of biological functions, especially in chromatin remodeling and epigenetic regulation^33–35^. To address the PfMORC localization, we first generated parasites containing a modified *pfmorc* locus, which incorporated the coding region for GFP at the 3′ end of the coding region (Fig. 1A,B) and referred as 3D7^*morc-GFP*^. Western blotting using anti-GFP antisera reveals that 3D7^*morc-GFP*^ expresses a fusion protein of approximately ~ 300 kDa protein while no signal was detected for control 3D7 parasites (Fig. 1C). Confocal imaging allows us to localize the protein and revealed a clear nuclear localization (based on colocalization with the Hoechst nuclear stain) in all asexual stages (Fig. 1D and Supplementary Fig. S1).

### Melatonin affects the expression of PfMORC in asexual stages

**Figure 2:**
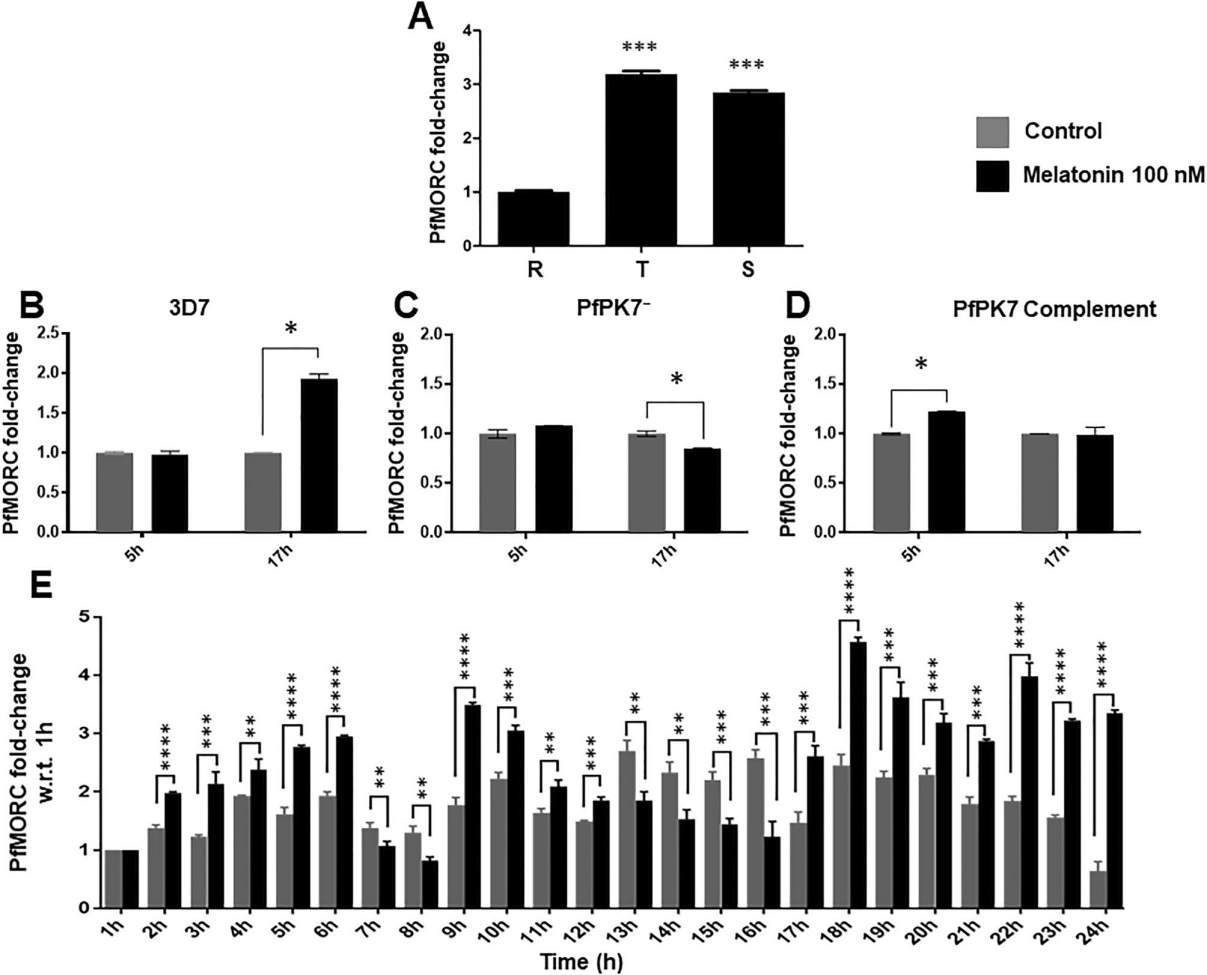
Real-time expression of PfMORC. (**A**) Reverse transcription real-time PCR (RT-qPCR) of PfMORC expression in different stages of 3D7 *P. falciparum* parasites. Stages—Ring (~ 10 h), Trophozoites (~ 30 h) and Schizonts (~ 42 h). (**B**) Validation by RT-qPCR of PfMORC expression in trophozoites stage (30 h post-infection) of 3D7; (**C**) PfPK7^−^ and (**C**) PfPK7 complement *P. falciparum* parasites. Trophozoites stage parasites were treated with 100 nM of melatonin for a period of 5 or 17 h. (**D**) Investigation of PfMORC expression pattern by RT-qPCR in 3D7 *P. falciparum* parasites. Ring stage parasites (6 h) were treated with 100 nM melatonin for a period of 24 h; the sample was collected every hour for the next 24 h period. The results were plotted as fold change in 1 h difference to the first collection. First, the relative expression was normalized with the reference gene (PF3D7_0717700) to obtain ΔC_t_ value and then with ΔC_t_ of 1 h control and melatonin treatment, respectively to obtain ΔΔC_t_ to calculate fold change in PfMORC expression. All the experiments were performed three times in triplicate and results are shown as mean ± SD. Statistical significance was performed by student t-test. *< 0.05.

We analyzed the transcriptional expression of PfMORC in the different morphological stages of the *P. falciparum* erythrocytic cycle. The ring stage real-time expression data was used as the basal for comparative analysis for the rest of the development stages. Figure 2A demonstrates that PfMORC is constantly expressed during the different blood stages, but the higher expression was observed in later developmental stages with approximately 3- and 2.7-fold higher in trophozoites and schizonts, respectively. We hypothesized that if this gene is involved in response to melatonin, then melatonin treatment may affect the expression of PfMORC. Our previous data suggested that melatonin treatment modulates the expression of genes associated with UPS, but this melatonin-dependent UPS down regulation is abrogated in a PfPK7^−^ line which lacks the orphan kinase PfPK7^22,24^. PfPK7 displays homology to MAPKKs C-terminal kinase domains and the N-terminal region is more closely to fungal protein kinase A (PKA) enzymes. No orthologues of PfPK7 has been reported in mammalian systems. Besides, a reduced number of merozoites per schizont was observed in PfPK7 knockout (PfPK7^−^) parasites suggesting a role of PfPK7 in parasite proliferation and development^36^. Based on the fact that PfPK7^−^ lines are non-responsive to melatonin, we then investigated the expression of PfMORC transcript in both wild type and PfPK7^−^ parasites after melatonin treatment to find the possible functional correlation of PfPK7 with PfMORC. We treated the synchronous ring stage *P. falciparum* wild type 3D7 and PfPK7^−^ parasites with 100 nM melatonin for 5 and 17 h, respectively. A housekeeping gene serine–tRNA ligase (PlasmoDB accession number: PF3D7_0717700) was used for initial normalization of PfMORC expression and then 5 h time point was used to compare the fold change. In wild type parasites, 5 h melatonin treatment did not affect PfMORC expression compared to the solvent control treatment. On the contrary, 17 h melatonin treatment showed approximately twofold elevated PfMORC expression compared to control treatment (Fig. 2B). On the other hand, when PfPK7^−^ parasites were treated with 100 nM melatonin for 5 h, the expression of PfMORC transcript was indistinguishable from that of controls, while after 17 h, the melatonin induced up-regulation of PfMORC transcript was significantly reduced (Fig. 2C). Complementing the *pfpk7* gene where PfPK7 is introduced back to the PfPK7^−^ parasites via an episome resulted in a slight increase in PfMORC transcript after 5 h melatonin treatment, but after 17 h the melatonin treatment significantly increased PfMORC mRNA expression, though not as efficiently as in wild type 3D7 parasites (Fig. 2D).

We have next examined the transcriptional trace of PfMORC in the intraerythrocytic cycle for 24 h period in *P. falciparum* wild type 3D7 parasites following melatonin treatment. Tightly synchronous 6 h post-infection (hpi) ring stage parasites were initially incubated with 100 nM melatonin and parasites samples were collected every hour during a 24 h period. The expression profile post melatonin treatment is shown in Fig. 2E, where both untreated and treated parasites for the first collection (1 h) are used as a standard to compare the difference in PfMORC fold change expression for control (untreated) and melatonin treatment respectively. As described previously, we normalize PfMORC with reference gene serine–tRNA ligase and then 1 h time point was taken to compare the fold change in PfMORC expression. The transcript of PfMORC showed a rhythmic profile throughout the 24 h period, with a peak at 18 h. The pattern in melatonin treated and control parasites was similar, but the expression level was lower in the untreated parasites and it started to decline after 20 h. Our combined data in Fig. 2 suggests that melatonin affects the abundance of PfMORC transcripts in Plasmodia during the intra RBC cycle.

### Knocking down PfMORC expression does not affect asexual growth of *P. falciparum*

**Figure 3:**
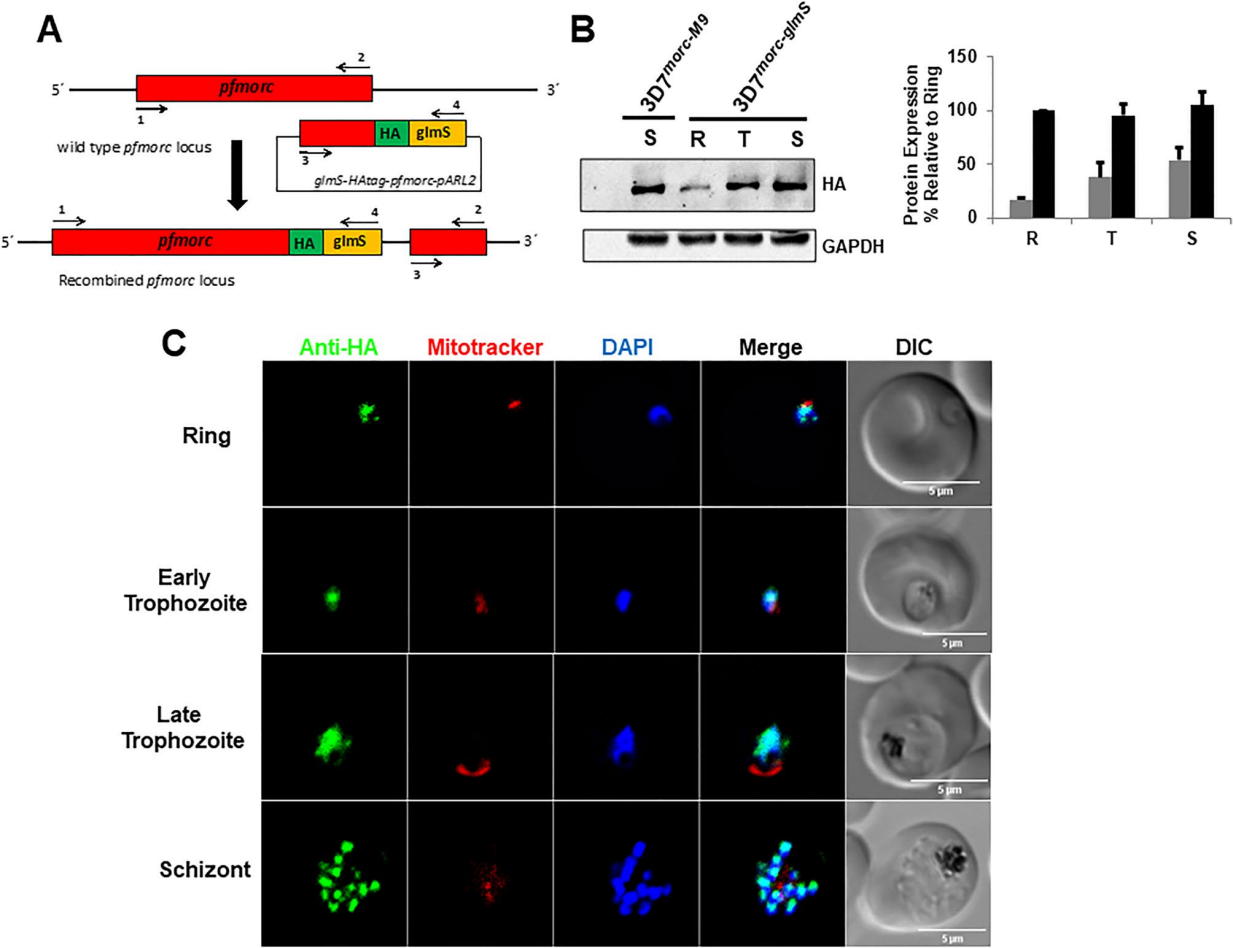
3D7^*morc-glmS*^ construct strategy and localization. (**A**) Schematic representation of the 3D7^*morc-glmS*^ integration strategy and gene after the integration; (**B**) Western blot of 3D7^*morc-glmS*^ parasites at different asexual stages showing different protein expression and wild type *P. falciparum* (3D7) as negative control showing no band (~ 300KDa) and (**C**) immunofluorescence assay and confocal microscopy to localize PfMORC in *P. falciparum*-infected erythrocytes (Ring, Trophozoite and Schizont Stage). Co-localization with nucleus stain DAPI confirms the GFP imaging that PfMORC localizes in the nucleus.

To investigate the potential role of PfMORC in the regulation of the *P. falciparum* asexual cycle, we used a glmS ribozyme based approach. The glmS ribozyme is found in many bacteria as a regulator of GlmS protein, and the self-cleaving activity of the ribozyme is enhanced in the presence of glucosamine-6-phosphate^37^. The glmS-ribozyme system has been previously used to induce the knockdown of the *P. falciparum* translocon of exported proteins (PTEX) and the antifolate drug target dihydrofolate reductase-thymidylate synthase (PfDHFR-TS)^38,39^*.* We engineered 3D7 parasites by incorporating a glmS ribozyme within the 3′-untranslated region of the *pfmorc* gene along with an additional triple hemagglutinin tag before the ribozyme. This parasite line was referred to as 3D7^*morc-glmS*^ (Fig. 3A). We also generated a control parasite line with the catalytically dead M19 version of glmS and referred to 3D7^*morc-M9*^ (Supplementary Fig. S2A). The Western blot using anti-HA antibodies with different asexual stage parasites revealed that the expression of PfMORC is higher in the late development stages (Fig. 3B and Supplementary Fig. S3B). We also confirmed, by immunofluorescence imaging of fixed 3D7^*morc-glmS*^ (Fig. 3C) and 3D7^*morc-M9*^ (Supplementary Fig. S2B), that the tagged protein is localized in the parasite nuclei (co-localizing with the nucleus stain DAPI).

**Figure 4:**
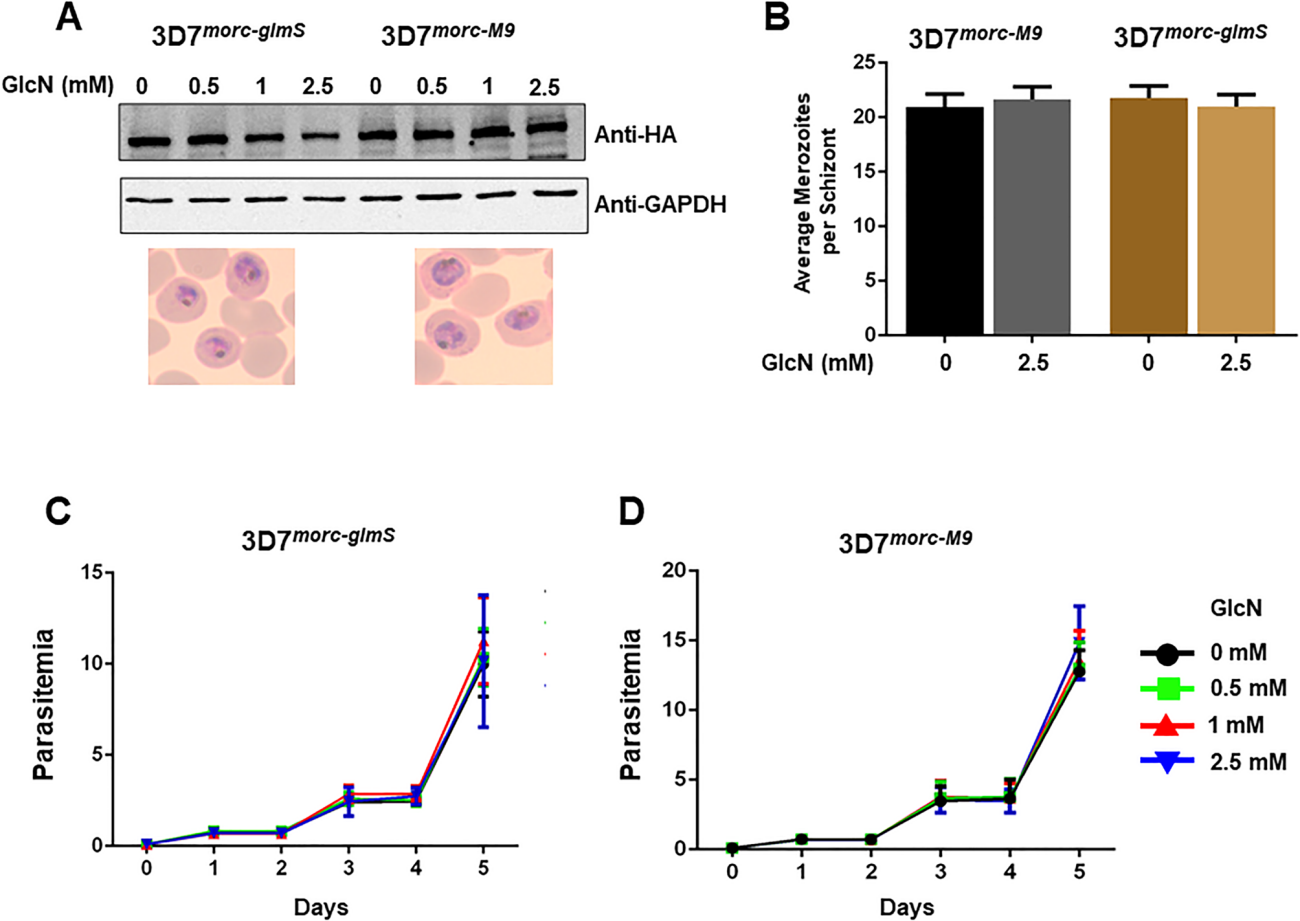
Effect of glucosamine treatment in conditional knockdown of PfMORC parasites. (**A**) Western blot analysis of 3D7^*morc-glmS*^ parasites treated with 0–2.5 mM of glucosamine for 48 h showing reduced protein expression, but the control 3D7^*morc-M9*^ parasites did not exhibit down regulation in protein level. (**B**) Early trophozoites were treated with and without 2.5 mM glucosamine till the next cycle and smears were made when majority of parasites reached to segmented schizont. The average of merozoites per schizont in populations was obtained by counting at least 50 parasites for each replicate. (**C**) Parasitemia was monitored for 6 days in the presence of a different concentration of glucosamine in the media for 3D7^*morc-glmS*^ and (**D**) 3D7^*morc-M9*^. No significant statistical difference was observed when unpaired t-test was performed.

We have next monitored the effect of the glmS ribozyme on PfMORC protein expression. The addition of glucosamine should result in cleavage and degradation of *PfMORC* mRNA, and a corresponding drop in the level of PfMORC protein. When 3D7^*morc-glmS*^ parasites were treated with 2.5 mM glucosamine for 48 h, the protein level was reduced by more than 50% compared to untreated controls. This reduction was not apparent in the similarly treated 3D7^*morc-M9*^ parasite line (Fig. 4A and Supplementary Fig. S4). From here on, we used 2.5 mM glucosamine concentration for all in vitro assays. To monitor potential morphological characteristics of the parasites conferred by PfMORC, these parasite lines were treated with 2.5 mM glucosamine at the early trophozoite stage and the number of merozoites per schizont was counted. Glucosamine treated and untreated parasites did not display any significant difference in terms of merozoites number per schizont (Fig. 4B). Similarly, we also monitored the parasite growth for 6 days in the presence of various concentrations of glucosamine. If gene knockdown has any effect on parasite growth, parasitemia should be reduced in a dose-dependent manner. Our result shows that knocking down PfMORC did not pose any detrimental effect on parasite growth and there was no significant difference in exponential growth even at higher (2.5 mM) glucosamine treatment (Fig. 4C) in 3D7^*morc-glmS*^. The control parasite line 3D7^*morc-glmS*^ was used as a negative control in the growth assay and also remained unaffected by the addition of glucosamine (Fig. 4D).

### Melatonin affects the parasite cell-cycle in 3D7^morc-glmS^ knockdown

**Figure 5:**
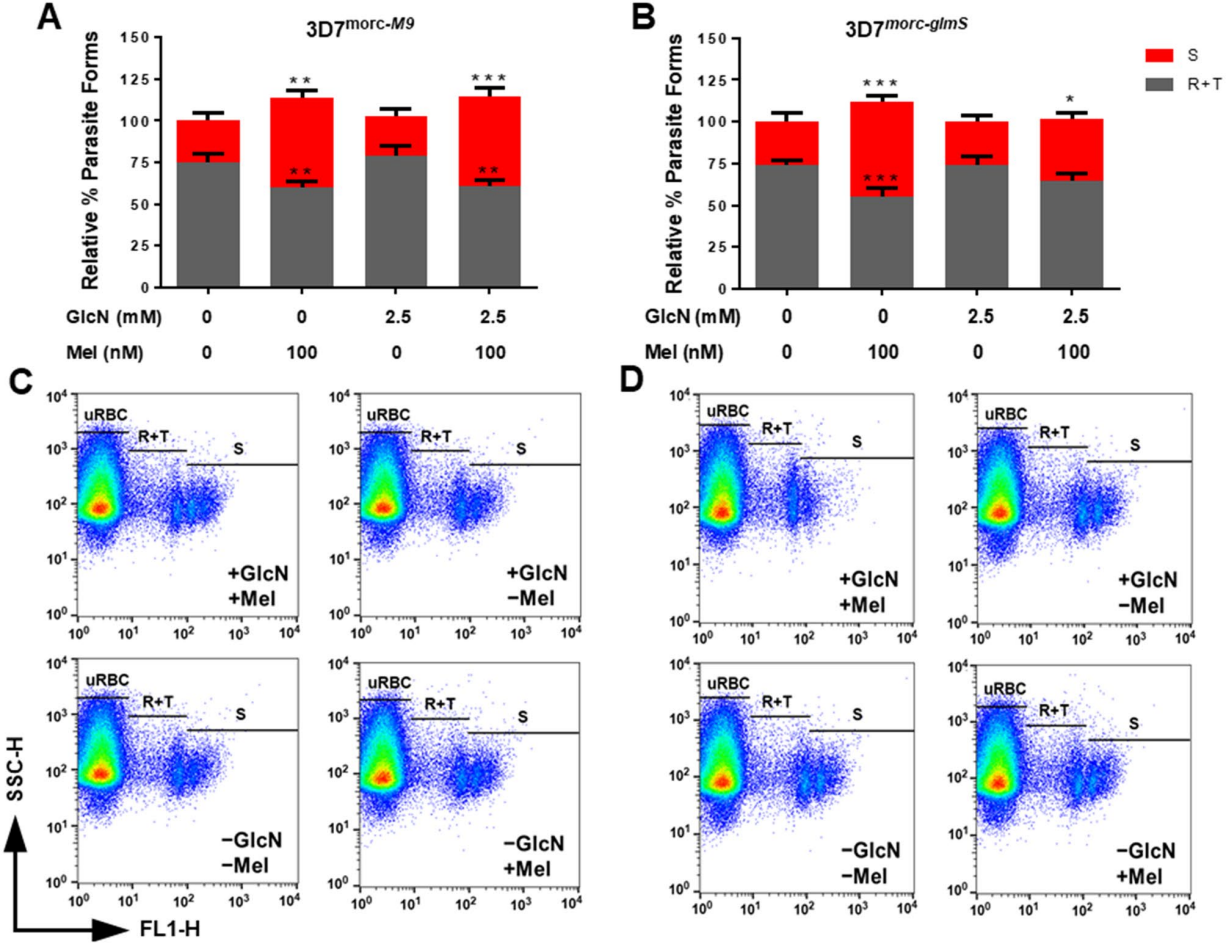
Effect of melatonin after knocking down PfMORC protein expression. Asynchronous parasites were first treated with or without 2.5 mM glucosamine for 48 h and then incubated with and without melatonin (100 nM) for 24 h. The parasites were stained with nuclear dye YOYO-1 and passed through the flow cytometer. R + T and S forms of the parasite population were calculated and plotted the percent population for both 3D7^*morc-M9*^ and 3D7^*morc-glmS*^. (**A**) Absence of melatonin shows high mono nucleated R + T form but 100 nM melatonin treatment increases the mature stage parasites in 3D7^*morc-M9*^ irrespective of glucosamine treatment; (**B**) but the effect of melatonin was partly interrupted to increase the S form 3D7^*morc-glmS*^, which is sensitive to glucosamine treatment, albeit R + T remained relatively similar to control. (**C**,**D**) and (**E**,**F**) represent the dot plot representation for melatonin effect with and without glucosamine for 3D7^*morc-M9*^ and 3D7^*morc-glmS*^, respectively. Each graphics represents the three independent experiments in triplicate for mean ± SD and the statistical difference was obtained by one-way ANOVA using Dunn’s multiple comparison test. *> 0.05, **> 0.01, ***> 0.001.

After 48 h with 2.5 mM glucosamine, the parasites were incubated for 24 h in the presence of 100 nM melatonin and we then monitored the parasite stage distribution. In Fig. 5A,B, the black bars of the histogram represent the rings and trophozoites (R + T) while red bars on top panel represent schizonts (S) parasites. We used an established protocol from our lab where parasites were stained with nuclear dye YOYO-1 and then separated the R + T (mono nucleated) and S (multi nucleated) forms by flow-cytometer^40^. The statistical comparison was done with respective R + T and S control where parasites were not treated with either glucosamine or nM melatonin.

Figure 5A depicts the percentage of R + T forms is approximately three times higher and S forms remained lower (approximately 25–30%) in 3D7^*morc-M9*^ parasites when they were not treated with melatonin regardless of glucosamine suggesting the insensitivity of the parasites for glucosamine. On the contrary, when melatonin treatment (100 nM) was done either with or without 2.5 mM glucosamine preincubation, regardless of glucosamine, S forms significantly increased to 45–50% and R + T was reduced to 50% (Fig. 5C). In 3D7^*morc-glmS*^ parasites, the absence of both glucosamine and melatonin did not affect parasite developmental forms, and the ratio of R + T versus S forms remained higher (Fig. 5B). This ratio, in the presence of melatonin, was modified without glucosamine and S forms elevated to; on the contrary, the addition of glucosamine partly abolished the effect of melatonin (Fig. 5D). In 3D7^*morc-glmS*^ parasites, the presence of glucosamine did not alter the R + T form significantly but the increase in S form is comparatively lower (approximately 35%) without glucosamine compare to melatonin control. Interestingly, the total parasitemia is approximately 10% higher in melatonin treated parasites relative to control but the presence of glucosamine shows a negative effect on this increase in 3D7^*morc-glmS*^ parasites (Supplementary Fig. S5). This result corroborates the previous reports where it was shown that melatonin treatment in wild type 3D7 parasites affects the in vitro intraerythrocytic cycle (Supplementary Fig. S6)^20^. The slight increase in parasitemia is because of more invasion events as a result of high S form when 100 nM melatonin was added to the culture. However, in the presence of glucosamine, melatonin treatment did not affect the multi/mono nucleated cell ratio.

## Discussion

We have shown that host hormone melatonin leads to parasite synchrony in the intraerythrocytic cycle through complex downstream signaling pathways in the human malaria parasite *P. falciparum*, as well as in the rodent *P. chabaudi*^20^. On the other hand, the non-synchronous rodent malaria parasites *P. berghei* and *P. yoelli* were unable to respond to melatonin^41^. Moreover, the rise in [Ca^2+^]_cyt_ and cAMP concentration^42^, as well as IP_3_ generation^19^ were involved in the cascade of melatonin signaling, although how these factors lead to the synchrony and whether they are coupled to other effectors are mechanistically unclear. Our previous work points to the central role of an atypical kinase PfPK7 in the downstream effect of melatonin synchronization and gene transcript as revealed by RNAseq expression profile differences in wild type compared with PfPK7 knock-out parasites^24^. Melatonin also activates a subset of genes related to the ubiquitin–proteasome system (UPS) in wild type *P. falciparum* parasites but not in PfPK7 knock-out parasites^22^. In the same direction, it was shown that parasites have the ability to exhibit rhythmic gene expression suggesting an intrinsic control^17,18^. However, using the murine model, it was demonstrated that in arrhythmic mice, synchrony of *P. chabaudi* was abrupted^17^ indicating the host cues have a role to play.

In a large scale PPI network study, PfMORC was identified as *microrchidia* family protein in the AP2-TFs interactome^31^. This protein has two conserve domains in the plasmodium species, first—kelch repeats and second—HATPase domain (Supplementary Fig. S7). The kelch superfamily of proteins contains the β-propeller tertiary structure and multiple protein–protein interaction sites to arbitrate different cellular functions, including gene expression, oxidative stress responses and ubiquitin-regulated protein degradation^43^. The second HATPase domain superfamily belongs to GHKL (gyrase, heat-shock protein 90, histidine kinase, MutL) family and is a typical characteristic of microrchidia (MORC) protein family^44^.

Our data demonstrate that melatonin treatment affects the expression of PfMORC transcript in PfPK7^−^ parasites. However, complementing PfPK7 partially recovered the loss it had in PfPK7^−^ knockout parasites. When we carried a time-dependent melatonin treatment in wild type *P. falciparum*, a wave-like or periodic PfMORC expression pattern was observed. The expression became constitutively higher after 18 h of the treatment suggesting the host hormone effect at peak during the later stage of the development.

We have directly localized PfMORC and our results with 3D7^*morc-GFP*^ parasites that constitutively fluorescence in the asexual stages indicated the nuclear localization of the protein. Recently, a combined approach of GeLC-MS/MS and 2D-LC–MS/MS has identified PfMORC in asexual nuclear fractions^32^. This report supports our subcellular localization results. It was reported that PfMORC is up-regulated in mefloquine resistant parasites when treated with mefloquine and also confers in cross-resistance to antimalarial halofantrine^45^. The protein microarray data identified PfMORC as highly immunogenic after the 6 months malaria season in the Mali region suggesting that PfMORC may also be associated with naturally acquired protection from uncomplicated malaria^46^. Interestingly, knocking down the PfMORC in 3D7^*morc-glmS*^ did not exhibit any difference in drug sensitivity against four tested drugs relative to control suggesting multiple factors may be involved in drug susceptibility (Supplementary Fig. S8). Our results suggest that PfMORC participates in parasite maturation and PfMORC knockdown with glucosamine alters the parasite synchrony with melatonin. We have for the first time identified and characterized PfMORC as an important *P. falciparum* protein that might be downstream to melatonin signaling pathways and mediates parasite response to this host hormone.

Our previous work shows the cytosolic counterparts of melatonin signaling such as the kinases PfPK7 and PKA as well as the ubiquitin–proteasome systems (UPS) machinery. Here we identified another component of the PfPK7 role in parasite synchronization by melatonin as the nuclear protein, namely PfMORC. How PfMORC leads to *P. falciparum* events remains to be elucidated. Interestingly, a global protein interaction profiling study identified 600 protein clusters spanning more than 20,000 proteins in the *Plasmodium* elucidate the potential PPI network of AP2-TFs along with MORC^31^. In another apicomplexan parasite *T. gondii,* MORC protein has been implicated to repress the sexual commitment via forming a complex with AP2-TFs and reruting the histone deacetylase protein TgHDAC3^47^.

It has been shown that members of AP2 proteins display a wide range of functions in both sexual and asexual proliferation of the parasite, although, approximately 80% of the AP2 proteins are expressed in the asexual cycle^15,16^. Considering the broad functional properties of AP2-TFs in the parasite, it could be possible that the interaction of PfMORC with an AP2-TF may have a possible regulatory mechanism in asexual cell cycle regulation. We have already known the role of melatonin in the synchronization of *P. falciparum* via cAMP—Ca^2+^ and UPS activation. These pathways are also linked with PfPK7 activation, which is also showing the differential expression of PfMORC upon melatonin treatment. Further, reducing PfMORC expression perturbs the cell cycle developmental stage strongly suggests the link between these components. Further studies are required to unravel the complex machinery parasites exhibit to survive inside the host erythrocytes.

## Methods

### *Plasmodium falciparum* culture

The *P. falciparum* strains (wild type 3D7 and PfMORC transgenics) were cultured at 37 °C in RPMI 1640 medium supplemented with 0.5% Albumax II (Gibco)^48^. Cultures were grown under 5% CO_2_, 5% O_2_, and 90% N_2_ atmosphere. The culture was synchronized with 5% sorbitol^49^.

### RNA extraction and real-time RT-PCR

Random-primed reverse transcription (RT) was performed using 1 µg of total RNA according to the SuperScript III kit protocol (Invitrogen). The relative levels of the transcripts were determined through quantitative PCR (qPCR) with Sybr Green PCR Master Mix (Applied Biosystem) using the 7300 Real-Time PCR System (Applied Biosystem). List of primers for PfMORC and Serine-tRNA ligase are provided in Supplementary Table S1.

### Transfection of malaria parasites

Transfection was performed as Waters et al.^50^. Pre warmed 370 µL (37 °C) cytomix (10 mmol/L K_2_HPO_4_/KH_2_PO_4_ (pH 7.6), 120 mmol/L KCl, 0.15 mmol/L CaCl_2_, 5 mmol/L MgCl_2_, 25 mmol/L Hepes (pH 7.6), 2 mmol/L EDTA) was added to the resuspended plasmid DNA (150 μg). Afterward, 200 µl *P. falciparum* 3D7 infected erythrocytes (~ 10% rings) were added and the mix was transferred into a cuvette. Following electroporation at 350 µF, 950 kV and high capacitance^51^, the transfected parasites were quickly inoculated together with 12 mL of pre-warmed complemented RPS (Gibco) and 400 µL fresh O^+^ blood. After 6 h, the WR with a final concentration 5 nM was added for positive selection of the transfected parasites. Media and antibiotic(s) were changed daily, alongside with the addition of 50 µL fresh O^+^ blood, until no more live parasites were observed in Giemsa stained smears. Then media and antibiotic(s) were changed in 4-day periods, alongside the addition of 50 µL fresh O^+^ blood, until Giemsa stains indicated parasite growth. List of primers and plastids used in *pfmorc* gene editing are listed in Supplementary Table S1.

### Glucosamine treatment

*Plasmodium falciparum* 3D7 constructs 3D7^*morc-glmS*^ and 3D7^*morc-M9*^ were synchronized with 5% sorbitol to achieve higher synchrony. Halfway of the parasite cycle which is 24 h post-invasion (hpi), the various concentration of glucosamine (0–2.5 mM) was added in the culture until the trophozoite stage of the next cycle (48 h) at 37 °C.

### Western blot

Saponin (0.05%) isolated parasites were resuspended in lysis buffer (in mM): 50 Tris, pH 8.0, 150 NaCl, 5 EDTA, and 0.5% Nonidet P40 in presence of protease inhibitor cocktail (Sigma). An equal amount of protein sample (25 µg) was loaded in each well and protein fractions were separated on SDS-PAGE gels, transferred to a nitrocellulose membrane, blocked with 5% milk, and probed with rabbit anti-GFP (1:2000), anti-HA (1:1500), anti-GAPDH (1:5000) and mouse anti-tubulin (1:5000) for overnight at 4 °C. After washing, the membrane was probed with horseradish peroxide-conjugated secondary anti-rabbit (1:30,000), anti-mouse (1:5000) antibody at room temperature for 1 h. The membrane was developed using 1 mL of ECL solution and 1 µL 30% H_2_O_2_.

### Immunofluorescence analysis of *P. falciparum*

Infected parasites were fixed for 30 min with 4% paraformaldehyde, 0.0075% glutaraldehyde in PBS. Cells were washed and permeabilized for 15 min with 0.1% Triton X-100 in PBS followed by washing. Fixed cells were then blocked with 3% BSA (in PBS) for 1 h at room temperature. Primary antibody labeling was done overnight at 4 °C with rabbit anti-HA (1:500) in PBS containing 3% BSA and 0.01% Triton X-100. Cells were washed and secondary antibody anti-IgG Rabbit Alexa 488 (Invitrogen) labeling was done at 1:300 dilutions in PBS containing 3% BSA for 1 h at room temperature. Cells were incubated for 5 min with DAPI 1:1000 and washed three more times in PBS. Cells were mounted using Vectashield (Vector). Images were acquired with a Zeiss confocal microscope (LSM 780-NLO) using UV and Argon 488 lasers. The main dichroic HFT UV (375) and 435–485 band-pass filters were used to collect DAPI fluorescence. The main dichroic HFT 488–514 band-pass filters were used to collect Alexa 488 fluorescence. The objective used was a plan-Neofluar 100×/1.3 with immersion oil. Images were analyzed by open sourced Fiji software^52^.

### Parasitemia and merozoite assessment

For parasitemia assessment, young trophozoites of 3D7^*morc-glmS*^ and ^*3D7morc-M9*^ were incubated at 0.1% parasitemia in 1 mL RPMI 1640 containing 0.5% albumax for 6 days in the presence of a different concentration of glucosamine. To estimate the growth progression, at least one thousand cells were counted on Giemsa stained slides prepared everyday.

To assess the number of merozoites in each schizont, a synchronized culture was maintained in RPMI containing 0.5% albumax and glucosamine until the next developmental stage when the majority of the parasites were segmented schizonts. From Giemsa stained slides, at least 50 schizonts were assessed in each slide. All these experiments were repeated 3 independent times in triplicate.

### Flow cytometry analysis

Asynchronous 3D7^*morc-glmS*^ and 3D7^*morc-M9*^ parasites were first treated with and without glucosamine for 48 h. After 48 h approximately 5% infected erythrocytes in 2% hematocrit were incubated with 100 nM of melatonin for 24 h. Parasites then were fixed in 2% formaldehyde in phosphate-buffered saline (PBS) for 24 h at room temperature and permeabilized with 0.1% Triton X-100 (Sigma-Aldrich) and 20 μg/mL RNAse (Invitrogen Life Technologies), incubated for 15 min at 37 °C and stained with 5 nM YOYO-1 (Molecular Probes) as performed by Schuck et al.^40^. Parasitemia and proportions of parasites at each stage were determined from dot plots [side scatter (SSC) versus fluorescence] of 10^5^ cells acquired on a FACS Calibur flow cytometer using CELLQUEST software (Becton and Dickinson). YOYO-1 was excited with a 488 nm Argon laser and fluorescence emission was collected at 520–530 nm. Initial gating was carried out with unstained, uninfected erythrocytes to account for erythrocyte autofluorescence. Stages of parasites were standardized with synchronized cultures.

### Statistical analysis

Statistical comparisons were made using either student’s t-test or one-way analysis of variance (ANOVA) followed by Dunn’s multiple comparison test using GraphPad Prism 5.01 (GraphPad Software Inc., San Diego, CA, USA). Experimental values are reported as the mean ± SD unless otherwise stated of at least three independent experiments performed on different days. Differences in mean values were considered significant at P < 0.05.

## Supplementary Information

Supplementary Information.
