# Supplementary material for: A nuclear protein, PfMORC confers melatonin dependent synchrony of the human malaria parasite P. falciparum in the asexual stage
Source: Sci Rep. 2021 Jan 21;11:2057. doi: 10.1038/s41598-021-81235-2 (PMC7820235; doi:10.1038/s41598-021-81235-2)
Supplement: Supplementary file 1 — Supplementary Information. [file 41598_2021_81235_MOESM1_ESM.pdf]

A nuclear protein, PfMORC confers melatonin dependent synchrony of the human malaria parasite *P. falciparum* in the asexual stage

Maneesh K. Singh[1,2], Giulliana Tessarin-Almeida[3], Barbara K. M. Dias[1,2], Pedro Scarpellli Pereira[1,2], Fahyme Costa[1], Jude M. Przyborski[4] and Celia R. S. Garcia [2*]

[1]Department of Parasitology, Institute of Biomedical Science, University of São Paulo, São Paulo, 05508-000, Brazil. [2]Department of Clinical and Toxicological Analyses, School of Pharmaceutical Sciences, University of São Paulo, São Paulo, 05508-000, Brazil. [3]Department of Physiology, Institute of Bioscience, University of São Paulo, São Paulo, 05508-090, Brazil. [4]Department of Biochemistry and Molecular Biology (BiMoBi), Interdisciplinary Research Center (iFZ), Justus-Liebig-University, GIESSEN, Germany.

Supplementary Figures

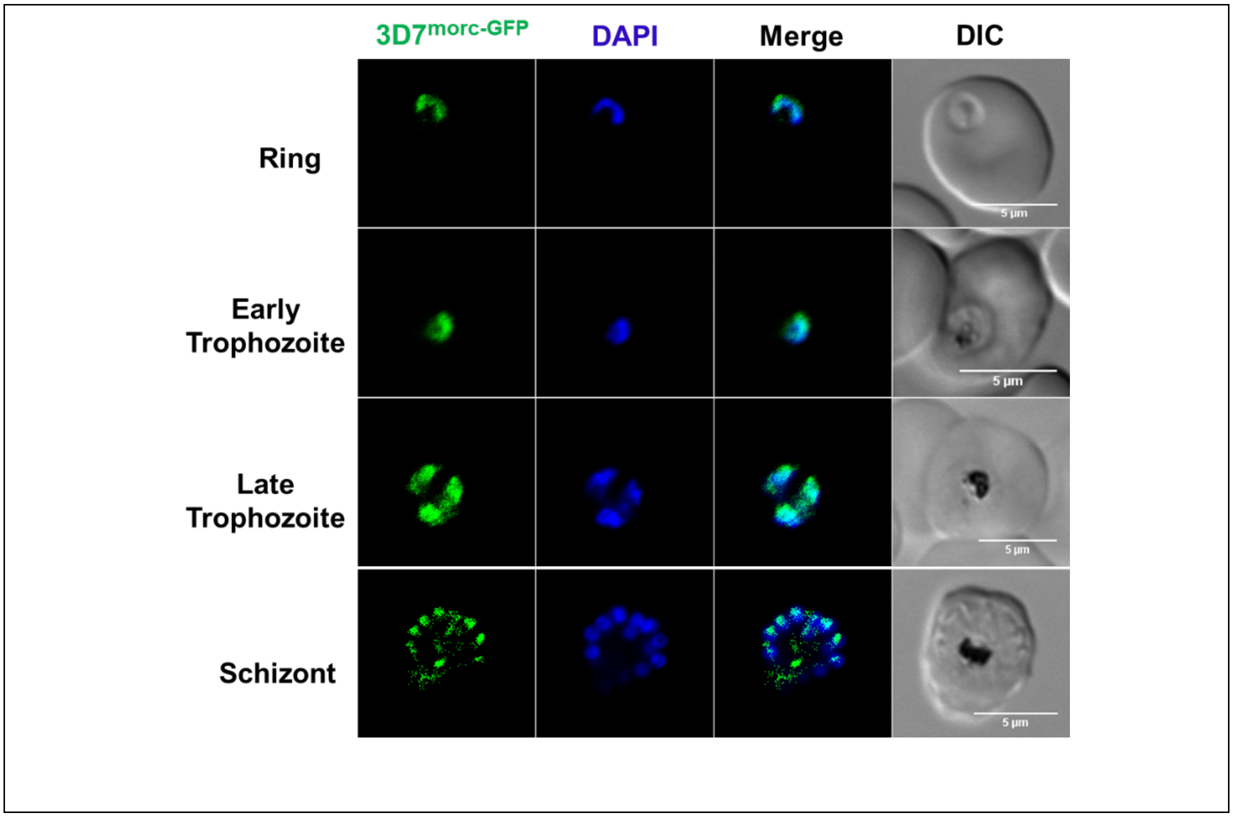

Supplementary Figure S1 – Confocal imaging of 3D7*morc-GFP*. Confocal microscopy of 3D7*morc-GFP* infected erythrocytes to localize PfMORC in different asexual stages. Parasites were collected at ~6-8 hpi (ring), ~20-24 hpi (early trophozoites), ~34-38 hpi (late trophozoites) and ~44-46 hpi (schizonts). Parasites were fixed, stained with a nuclear dye DAPI and mounted on a clean glass slide to perform imaging. Co-localization with nucleus stain DAPI shows the PfMORC localizes in the nucleus.

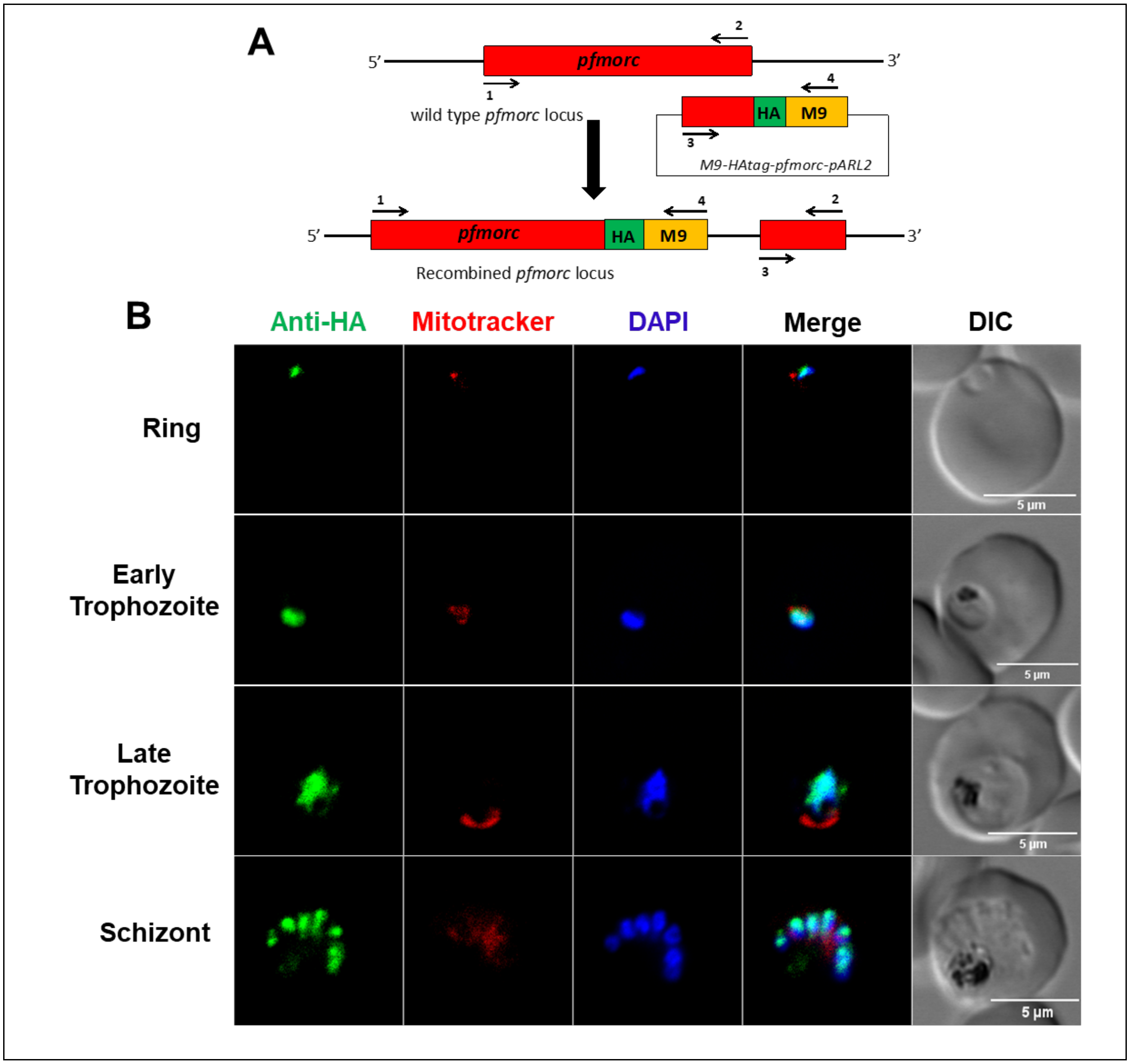

Supplementary Figure S2 - 3D7$^{morc\text{-}M9}$ construct strategy and localization. (A) Schematic representation of the 3D7$^{morc\text{-}M9}$ integration strategy and gene after the integration; **(B)** Immunofluorescence assay and confocal microscopy to localize PfMORC in *P. falciparum*-infected erythrocytes (Ring, Trophozoite and Schizont Stage). Co-localization with nucleus stain DAPI shows the PfMORC localizes in the nucleus.

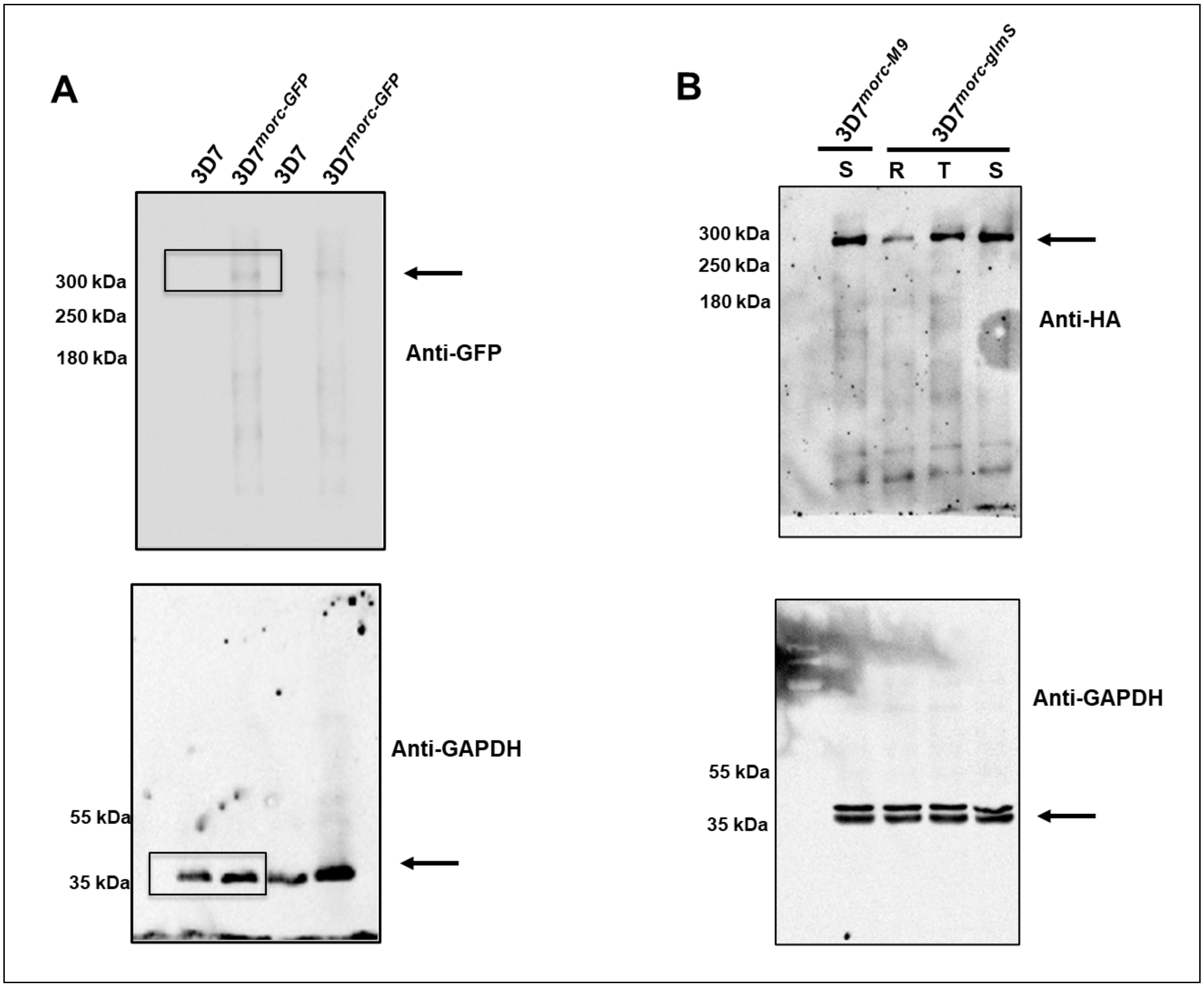

Supplementary Figure S3 - Full-length Western blot analysis of **(A)** 3D7$^{morc\text{-}GFP}$ and **(B)** 3D7$^{morc\text{-}glmS}$. Anti-GAPDH control to the bottom of each blot indicates equal loading.

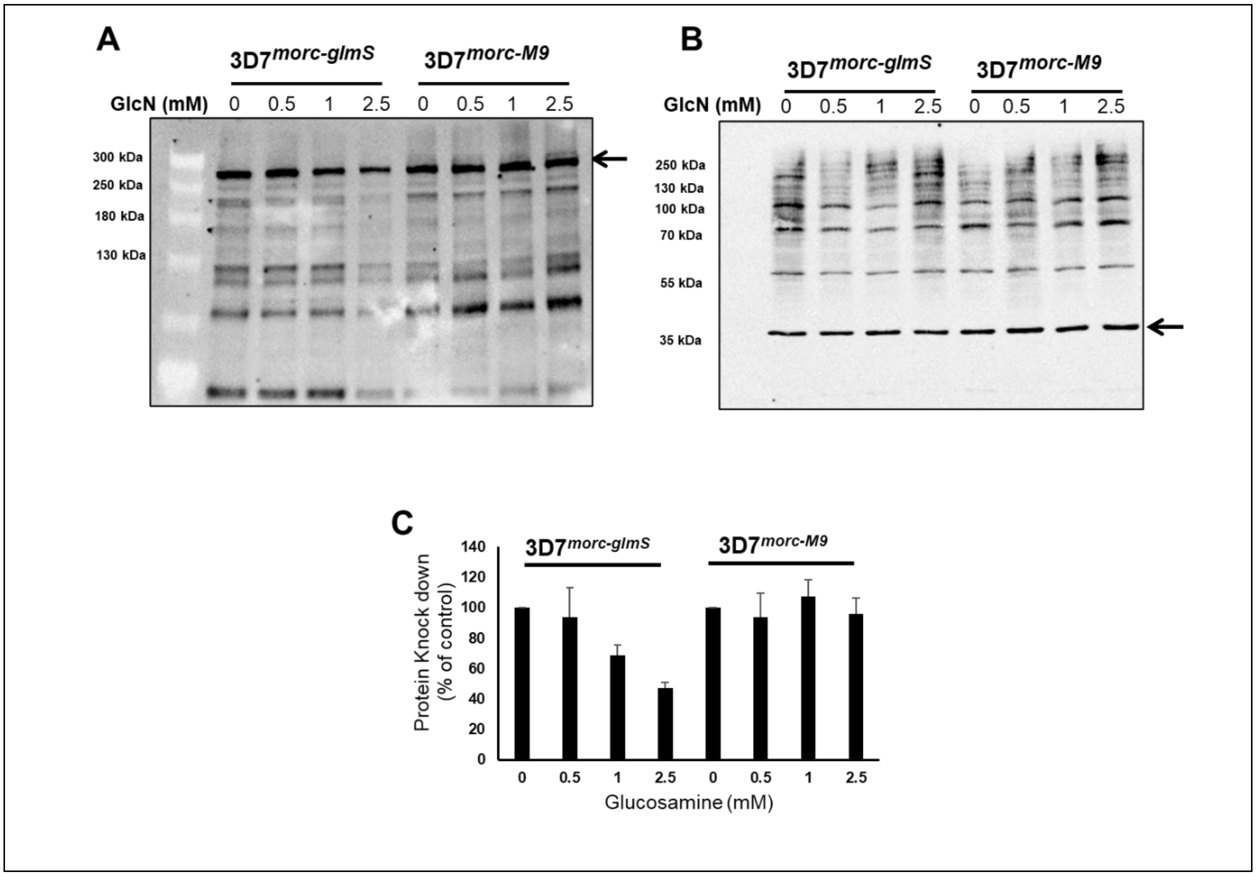

Supplementary Figure S4 – Full-length Western blot analysis of 3D7$^{morc\text{-}glmS}$ **(A)** parasites treated with 0-2.5 mM of glucosamine for 48 h showing reduced protein expression, but the control 3D7$^{morc\text{-}M9}$ parasites did not exhibit down regulation in protein level. **(B)** Anti-GAPDH in the right panel indicates equal loading which is also depicted in the bottom where band densitogram **(C)** was performed.

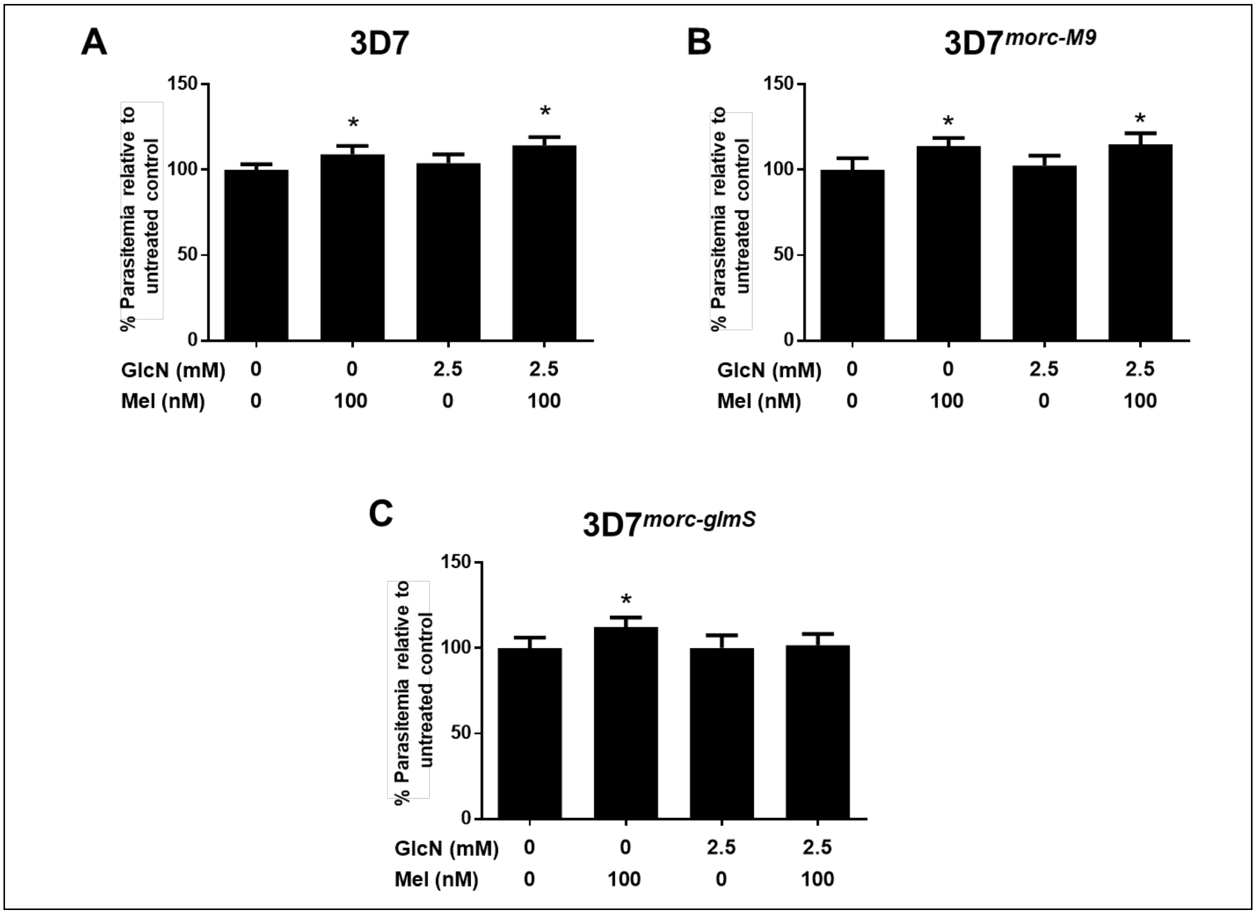

Supplementary Figure S5 – Total parasitemia relative to control for 3D7 **(A)**, 3D7$^{morc\text{-}M9}$ **(B)**, and 3D7$^{morc\text{-}glmS}$ corresponding to Figure 5. Only control strains showing the change in total parasitemia after melatonin treatment regardless of glucosamine preincubation **(A & B)**. However, 3D7$^{morc\text{-}glmS}$ has increase parasitemia after melatonin only in absence of glucosamine but this change was not noticeable when glucosamine was present **(C)**.

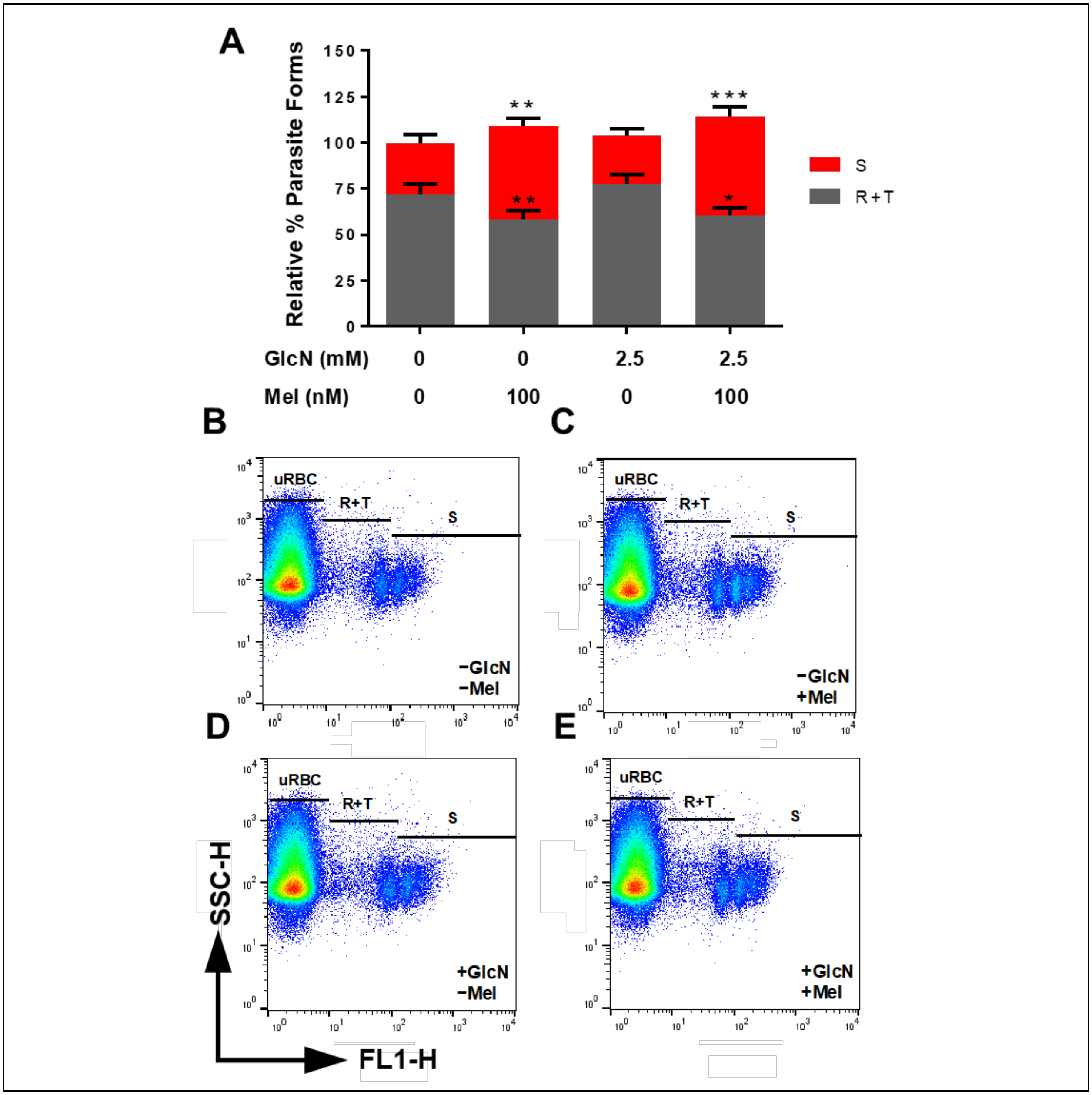

Supplementary Figure S6 – Effect of melatonin in wild-type 3D7 parasites. Asynchronous parasites were first treated with or without 1 mM glucosamine for 48 h at the early trophozoite stage. After 48 h, parasites were induced with and without melatonin (100 nM) for 24 h. Melatonin treatment increases the mature stage parasites in 3D7 irrespective of the glucosamine treatment since 3D7 parasites lack glmS ribozyme **(A, B & D)**. However, parasites without melatonin were unable to show the fast maturation as shown in the histogram and dot-plot graphics **(A, C & E).** Each graphics represents the three independent experiments in triplicate and the statistical difference was obtained by unpaired t-test. * >0.05; ** >0.01; *** >0.001

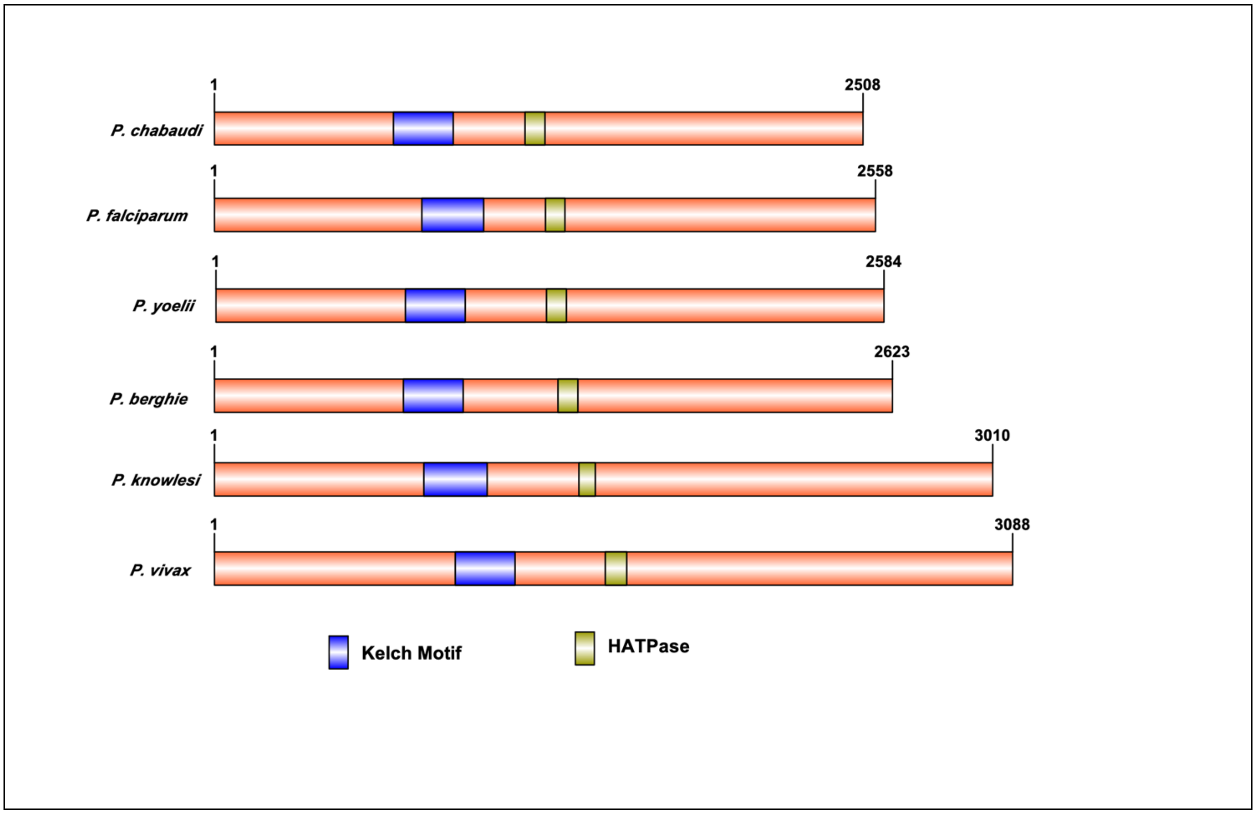

Supplementary Figure S7 – Schematic diagram of PfMORC protein and domain homology using BLAST search. Domain architecture of full-length PfMORC protein was generated using the InterPro program showing the kelch-like motif and a HATPase domain.

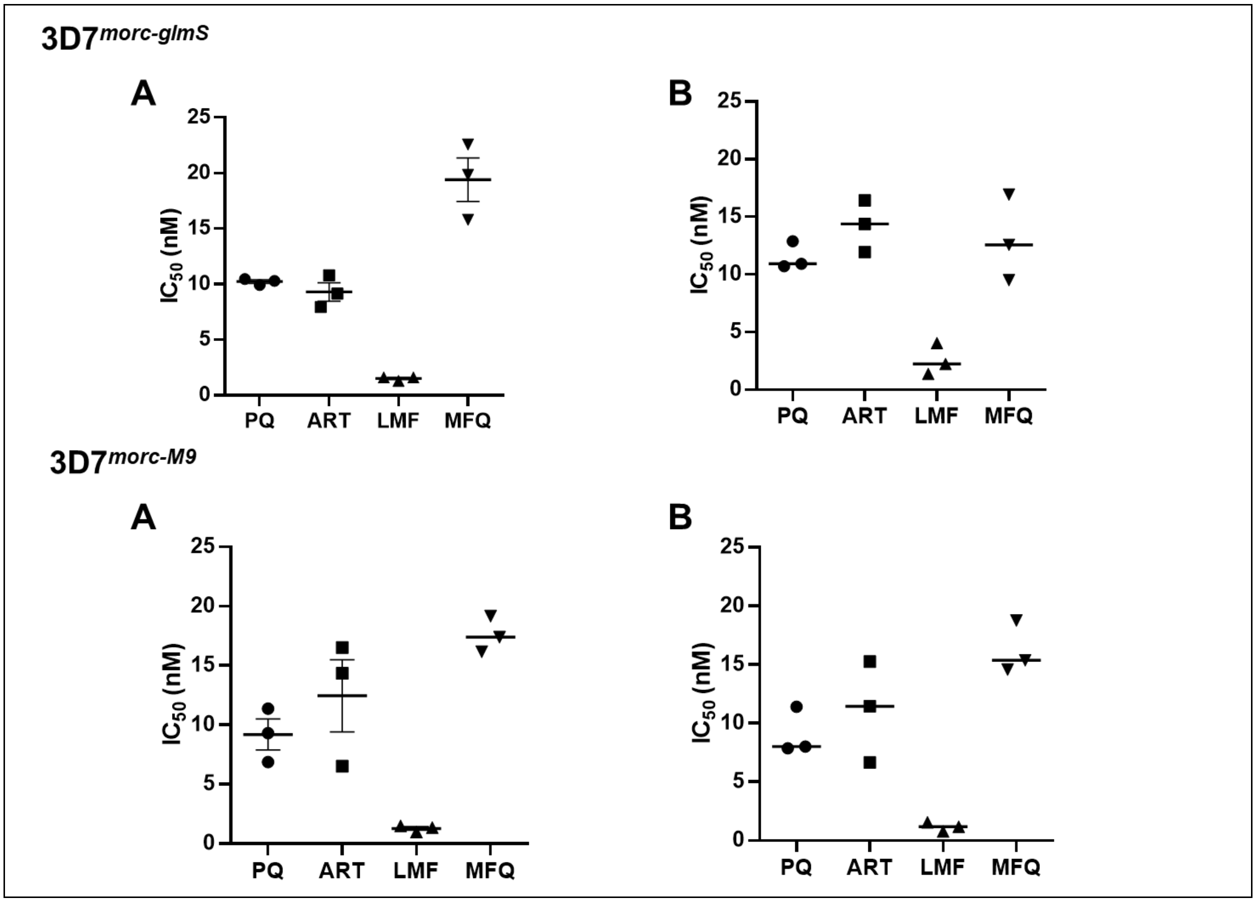

Supplementary Figure S8 - Effect of antimalarial drugs in PfMORC transgenic parasites. Parasites were first treated with and without 2.5 mM GlcN for 48 h and then 0.3 % parasitemia in 2 % hematocrit was incubated with serially diluted drugs (PQ – piperaquine; ART – artemisinin; LMF – Lumafantrine, and MFQ – mefloquine) for 72 h. Dual stained parasites with mitotracker (Invitrogen) and sybr green (Invitrogen) were counted in Flow Cytometer [53]. IC50 values were calculated for 3D7$^{morc\text{-}glmS}$ without GlcN treatment **(A)**, with GlcN treatment **(B)** and for 3D7$^{morc\text{-}M9}$ without GlcN treatment **(C)**, with GlcN treatment **(D)**.

Supplementary Table S1 - List of oligonucleotides and plasmids used in this study

**Primer**	**Sequence 5´→ 3´**	
MORC_Fwd	AGCACAACACAAATGCCACT	used in Real-Time RT-PCR
MORC_Rev	AGCGAAAGCTCCAAAGAGCC	
Serine-tRNA ligase_Fwd	TGGAACAATGGTAGCTGCAC	
Serine-tRNA ligase_Rev	TCATGTATGGGCGCAATTT	
PfMORC Fwd (1)	AAATGAAAATGAAGAGAAACCAAATGATGG	used to edit the *pfmorc* locus
PfMORC Rev (2)	GATGCCGAGGAGGAAGATACCATA	
Not_70 F (3)	GGCGGATAACAATTTCACACAGG	
PARL_Rev (4)	CAGTTATAAATACAATCAATTGG	
**Description**	***E. coli* resistance marker**	***P. falciparum* resistance marker**
pARL2- GFP	Amp^R	WR99210
pARL2- glmS	Amp^R	WR99210
pARL2- glmS-HAtag	Amp^R	WR99210
pARL2- M9	Amp^R	WR99210
pARL2- M9-HAtag	Amp^R	WR99210
